# Staged Starnes Operation Preserving Patent Ductus Arteriosus for Neonates with Ebstein’s Anomaly and Pulmonary Atresia

**DOI:** 10.2174/157340308784245829

**Published:** 2008-05

**Authors:** Yoichi Kawahira, Kyoichi Nishigaki, Hideto Ozawa, Tsugutoshi Suzuki

**Affiliations:** 1 Department of Pediatric Cardiovascular Surgery, Osaka City General Hospital, 2-13-22 Miyakojimahondori, Miyakojimaku, Osaka-city, Osaka, 534-0021, Japan; 2 Department of Pediatric Cardiology, Osaka City General Hospital, 2-13-22 Miyakojimahondori, Miyakojimaku, Osaka-city, Osaka, 534-0021, Japan

**Keywords:** Starnes operation, patent ductus arteriosus, Ebstein’s anomaly, pulmonary atresia, arrhythmia surgery.

## Abstract

We herein reported 2 successful neonates with Ebstein’s anomaly and small pulmonary arteries undergoing Starnes operation preserving the patent ductus arteriosus. Subsequent Blalock-Taussig shunt was carried out 1 or 2 months after the first surgery. One case had already undergone a successful Fontan operation, and the other had a successful bidirectional Glenn shunt so far. This staged Starnes strategy might be a safe and simple choice for neonates with Ebstein’s anomaly and small pulmonary arteries.

## INTRODUCTION

Starnes *et al*. have reported a new surgical technique consisting of closure of the tricuspid valve and placing an aortopulmonary shunt for neonates with Ebstein’s anomaly [1]. However, size of the pulmonary arteries of these neonates is sometimes extremely small, reflecting their small amount of pulmonary blood flow with the immature lungs [2]. In this situation, postoperative pulmonary blood flow might not be adequate.

We have carried out Starnes operation preserving patent ductus arteriosus (PDA) to obtain adequate pulmonary blood flow after the surgery in 2 neonates with Ebstein’s anomaly associated with small pulmonary arteries.

## CASE 1

A 1-day-old neonate, diagnosed as Ebstein’s anomaly and pulmonary atresia by fetal echocardiography, was transferred to our center due to cyanosis. Chest X-ray on admission showed cardio-thoracic ratio of 96%. Echocardiography showed the huge right atrium and right ventricle, and that the septal and the posterior leaflet of the tricuspid valve displaced downwards. The pulmonary artery was very small. Starnes operation was carried out 12 days after birth.

**Fig. (1):**
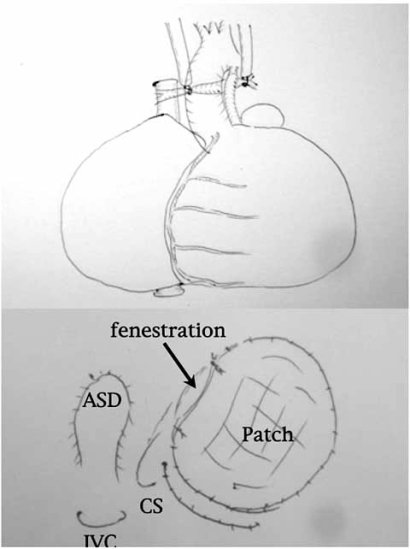
Schema of the first-stage operation indicating both pulmonary arteries snared to establish cardiopulmonary bypass (upper), and partial closure of the tricuspid valve without stitches near the atrio-ventricular node (lower).

At the operation, cardiopulmonary bypass was established with standard aortic and bicaval cannulation. The size of the bilateral pulmonary arteries were 2mm and 2.5mm, respectively. We gave up aortopulmonary shunting to the pulmonary artery with Starnes operation, and decided to go for preservation of PDA. After the bypass was established, bilateral pulmonary arteries were snared down to stop pulmonary blood flow without touching PDA (Fig. **1**). After induction of cardioplegic arrest, the atrial septal defect was enlarged at first. Next the true annulus of the tricuspid valve was partially closed using Dacron patch. We did not put any sutures near the atrio-ventricular node (AVN) to avoid injury of AVN and to make a small communication between the right atrium and the right ventricle. The right atrium was plicated at last. After both pulmonary arteries were declamped to supply the lungs through PDA, patient was weaned from the bypass. That was not difficult.

PGE1 was postoperatively infused to remain PDA open and to keep stable hemodynamics for 19 days. Dose of PGE1 was changed from 3 ng/kg/min to 16 ng/kg/min according to the systemic arterial oxygen saturation. Then right modified Blalock-Taussig shunt was carried out using 4mm ePTFE tube.

Postoperative chest X-ray showed 65% of CTR, and echocardiography showed the small right atrium and the right ventricle and the enlarged left ventricle. She successfully underwent total cavo-pulmonary connection at the age of 1 year, and is doing well now.

## CASE 2

A baby, who was diagnosed with Ebstein’s anomaly, pulmonary atresia, and recurrent paroxymal supraventricular tachycardia by fetal echocardiography, was delivered by cesarean section in our center. Birth weight was 2678g. Apgar score was 7/8. PSVT had occurred after his birth and required infusion of procaineamide. Chest X-ray showed CTR of 86%. Echocardiography demonstrated the large right atrium, the septal and posterior leaflet of the tricuspid valve displacing downwards, and the left vetricle compressed by the large right ventricle. The bilateral pulmonary arteries were 2.5 mm and 2.0 mm in diameter, respectively. Then, low cardiac output had gradually progressed. We decided to go for Starnes operation and cryoablation for this 4-day-old baby.

At the operation, electro-physiological mapping was performed under a beating heart at first. During ventricular pacing, the earliest atrial activity was identified between the coronary sinus and AVN with the decremental conduction. Arrhythmia repeatedly occurred preoperatively was diagnosed as atrio-ventricular node reentrant tachycardia, and perinodal cryoablation was carried out.

Cardiopulmonary bypass was instituted with standard aortic and bicaval cannulation. Both pulmonary arteries were clamped to stop pulmonary blood flow. After cardiac arrest, perinodal cryoablation was done at first. Next, the atrial septal defect was enlargerd, and the tricuspid valve was partially closed with dacron patch not to injury AVN (Fig. **1**). The right atrium was extensively plicated. Weaning from the bypass was not difficult with continuous infusion of PGE1. The both Pulmonary arteries were declamped to recover pulmonary blood flow from PDA with a stable hemodynamics. Systemic arterial oxygen saturation was about 80% with systolic blood pressure of 60 mmHg.

She was extubated 7 days after the surgery with a stable condition. Postoperative dose of PGE1 had remained 5 ng/kg/min for 2 months with systemic arterial oxygen saturation of 80% to 85%. The right modified Blalock-Taussig shunt was safely carried out 2 month after the surgery. Bidirectional Glenn shunt was successfully carried out at the age of 1 year. Now she is waiting for Fontan procedure with a good condition.

## DISCUSSION

A new palliative open-heart surgery for neonates with Ebstein’s anoamaly has been reported in 1991, which consisted of exclusion of the right ventricle and establishment of an aortopulmonary shunt [1]. This surgery still has poor outcomes mainly due to left ventricular dysfunction [2,3]. Another problem we have often experienced was inadequate pulmonary blood flow after the operation, which might result from the very small pulmonary arteries with a high vascular resistance of this entity. Aortopulmonary shunting to these neonates with small pulmonary arteries would not technically easy to be done, and a major risk. In this situation, reestablishment of another shunt might be sometimes needed during the surgery or immediately after the surgery. We decided to preserve PDA as a pulmonary blood supply at the Starnes operation, and, a few months later, to carry out Blalock-Taussig shunt as a second staged procedure for these neonates with small pulmonary arteries.

Although this first-stage procedure preserving PDA requires continuous infusion of prostaglandin E1 postoperatively, this strategy could allow the first Starnes operation to be much safer and simpler. In our 2 cases, postoperative SpO2 remained stable with oxygen saturation of 78% to 88% with use of PGE1. At the modified Blalock-Taussig shunt carried out 1 or 2 months after the surgery, their pulmonary artery got larger and patient’s stability was much high.

To control postoperative pulmonary blood flow by PGE1 was not difficult. In case 1, the dose was changed from 5 ng/kg/min to 16 ng/kg/min postoperatively, however the dose in case 2 had remained same dose of 5 ng/kg/min with SpO2 of 80%’s. When we can not control the adequate pulmonary flow, we would have to go for early systemic-pulmonary shunt.

Arrhythmia surgeries for 3 neonates with congenital heart diseases associated with WPW syndrome or atrial tachycardia have been reported by Mavroudis *et al.* [4]. In this entity with recurrent tachycardia, postoperative tachy-arrhythmia is a life-threatening major problem deteriorating their hemodynamics. Even in the neonates, attempt of intraoperative mapping indicating the earliest atrial activity might be feasible because this mapping could be simply carried out under the beating heart. Our 6-day-old neonate successfully underwent perinodal cryoablation with no postoperative recurrence of the tachy-arthythmia.

Staged Starnes strategy consisting of the first-stage Starnes operation preserving PDA and a subsequent modified Blalock-Taussig shunt as a second procedure might be a good option for neonates with Ebstein’s anomaly associated with small pulmonary arteries.

## ABBREVIATIONS

ASD: = Atrial septal defect
CS: = Coronary sinus
Patch: = Xenopericardial patch
IVC: = Inferior caval vein
Fenestration: = Gap between the true annulus and patch near the atrio-ventricular node
